# Choroidal thickness using EDI-OCT in adult-onset vitelliform macular dystrophy

**DOI:** 10.1186/s40942-016-0031-1

**Published:** 2016-02-05

**Authors:** Renato Menezes Palácios, Thaís Sousa Mendes, Ronaldo Yuiti Sano, Davi Chen Wu, Teruo Aihara, Roberta Pereira de Almeida Manzano

**Affiliations:** 1 Department of Ophthalmology - Retina, Irmandade da Santa Casa de Misericordia de São Paulo, Rua Martinico Prado, 159 - apto 63, São Paulo, SP CEP 01224-010 Brazil; 2 Ophthalmology, Santa Casa de Sao Paulo School of Medical Sciences, São Paulo, SP Brazil

**Keywords:** Choroidal thickness, Adult-onset foveomacular vitelliform dystrophy, Optical coherence tomography, Enhanced depth imaging

## Abstract

**Background:**

To compare choroidal thickness in patients with adult-onset foveomacular vitelliform dystrophy (AOFVD) with healthy subjects and to correlate choroidal thickness with age, gender and spherical equivalent.

**Methods:**

A prospective, observational study of 37 eyes (15 eyes in AOFVD group and 22 eyes in control group) was conducted. Images were acquired by enhanced depth imaging optical coherence tomography (EDI-OCT). Choroidal thickness measurements were performed in the subfoveal region and at 500, 1000 and 1500 µm intervals from the foveal center to nasal and to temporal regions for subsequent averaging of values.

**Results:**

The AOFVD group consisted of four male eyes (28.6 %) and 10 female eyes (71.4 %); age was 33–62 years; spherical equivalent (SE) ranged from −1.50 to 1.50 spherical diopters (SD); mean subfoveal thickness was 325.6 µm, ranging from 186 to 420 µm; and the average of thicknesses was 309.4 µm, ranging from 188 to 413 µm. The control group consisted of 12 male eyes (54.5 %) and 10 female eyes (45.5 %); age was 27–62 years; SE ranged from −2.50 to 0.50 SD; subfoveal thickness was 294.8 µm, ranging from 213 to 481 µm; and the average of thicknesses was 279.4 µm, ranging from 201 to 458 µm.

**Conclusions:**

The AOFVD group and the control group showed similar choroidal thickness by correcting for age, SE and gender. Not yet known, completely, which biochemical and vascular flow alterations of the choroid, and which functional RPE changes may play a role in the pathogenesis of this disease. EDI-OCT, incorporated in some SD-OCT devices, allows higher quality assessment of the choroid. In this article, choroidal thickness of patients with AOFVD, a rare disease with a not fully understood pathogenesis, was assessed.

## Background

Adult-onset foveomacular vitelliform dystrophy (AOFVD) was first described in 1974 by Gass [1–3]. AOFVD typically occurs between the fourth and sixth decades of life and has sporadic or autosomal dominant inheritance, with variable expression and incomplete penetrance [1, 3, 4]. The histopathological characteristics of the disease have yet to be fully established. The condition involves the accumulation of yellowish, heterogeneous, symmetric, rounded or oval material, located in the macular region between the photoreceptive layer and the retinal pigment epithelium (RPE) [1, 2, 4]. In early stages, patients are generally asymptomatic. As the disease progresses, slow and progressive visual loss occurs. Some rare complications may also appear such as subretinal neovascular membrane [1].

The choroid plays an important role in ocular physiology, providing metabolic support to the RPE and external retina. The choroid can be affected in a number of diseases, such as age-related macular degeneration (ARMD), polypoidal choroidal vasculopathy (PCV), central serous chorioretinopathy (CSC), pathological myopia, choroidal melanoma, as well as atherosclerotic microvascular disease and inflammatory processes [2, 5, 6].

Currently, optical coherence tomography (OCT) is used for the study of many different retinal diseases. Following the introduction of enhanced depth imaging (EDI-OCT) technique in spectral domain OCT (SD-OCT) devices, a growing number of researchers have studied choroidal thickness in healthy eyes and in various diseases to correlate changes with pathogenesis. EDI-OCT allows better image quality and more accurate assessment of the choroidal morphology [2, 7–9].

The main purpose of this paper was to compare choroidal thickness of AOFVD patients with healthy subjects. Furthermore, the secondary purpose was to correlate choroidal thickness with age, sex and spherical equivalent (SE).

## Methods

The present prospective, observational study was conducted at the Department of Ophthalmology of the Santa Casa de Misericórdia de São Paulo Hospital between December, 2014, and September, 2015.

**Fig. 1:**
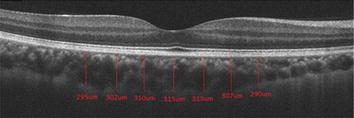
EDI-OCT of a subject from control group. Manual measurements were performed in the subfoveal region and at 500, 1000 and 1500 µm intervals from the foveal center to nasal and to temporal regions. Choroidal thickness was defined as the distance between the outer border of the RPE and the hyper-reflective line corresponding to the border between choroid and sclera

**Fig. 2:**
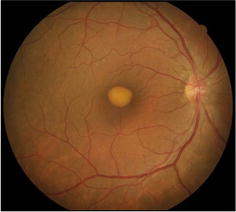
Retinography of a patient from AOFVD group. Classic pattern on retinography of a patient with AOFVD, revealing accumulation of yellowish, heterogeneous, rounded or oval material located in the macular region

The study followed the principles of the Declaration of Helsinki. The nature and possible consequences of the study were explained and all of the participants subsequently signed the informed consent. All participants were submitted to a complete ophthalmologic exam, EDI-OCT (Fig. 1), retinography (Fig. 2) and autofluorescence. In some patients, the electrooculogram (EOG) exams had been documented in another hospital prior to this study.

Exclusion criteria were: “presence of significant media opacity”, “amblyopia”, “refraction error [SE outside the −2.50 to +2.50 spherical diopters (SD) range]”, “history of retinal or choroid disease”, “history of any intraocular surgery or photodynamic or focal laser therapy or treatment involving intravitreal injection of anti-VEGF/corticosteroids”.

The EDI-OCT (Cirrus HD-OCT ^®^, Carl Zeiss Meditec, Dublin, CA) of image acquisition, by using ocular tracking technology, enhances the acquisition and quality of images, although no automatic measurement function is available in the software. Image generation was achieved using 1 line raster, consisting of 6 mm lines corresponding to 4096 A-scans [7, 8]. The intensity of the signal chosen was at least 8 (total of 10). Choroidal thickness was defined as the distance between the outer border of the RPE and the hyperreflective line corresponding to the border between choroid and sclera.

Manual measurement of choroidal thickness was performed using the caliper tool. Measurements were performed in the subfoveal region and at 500, 1000 and 1500 µm intervals from the foveal center to nasal and to temporal regions to determine the average of the values. The measurements were made and analysed by two researchers. In the presence of discrepancy (over 20 µm), another measurement was made by both.

A *p* value lower than 0.05 (5 %) was considered statistically significant (Spearman’s correlation coefficient, Pearson’s Chi square, and Mann–Whitney). All statistical analyses were performed using version 2.15.2 of the R Statistics program.

## Results

This study included 36 eyes (18 individuals), including 14 eyes (40.5 %) in AOFVD group and 22 eyes (59.5 %) in control group. Both eyes of all participants were included.

The AOFVD group consisted of four male eyes (28.6 %) and ten female eyes (71.4 %). AOFVD patients had a mean age of 46.3 years, range 33–62 years, with a standard deviation of 11.3 years. Mean SE was 0.04 SD, ranging from −1.50 to 1.50 SD, with a standard deviation of 1.05 SD. In AOFVD group, mean subfoveal thickness was 325.6 µm, ranging from 186 to 420 µm, with a standard deviation of 65.5 µm. The average of thicknesses was 309.4 µm, ranging from 188 to 413 µm, with a standard deviation of 56.7 µm.

The control group consisted of 12 male eyes (54.5 %) and 10 female eyes (45.5 %). Control group had a mean age of 32.7 years, range 27–62 years, with a standard deviation of 9.7 years. Mean SE was −1.00 SD, ranging from −2.50 to 0.50 SD, with standard deviation of 1.11 SD. Mean subfoveal thickness of controls was 294.8 µm, ranging from 213 to 481 µm, with standard deviation of 70.3 µm. The average of thicknesses was 279.4 µm, ranging from 201 to 458 µm, with a standard deviation of 66.1 µm.

The results revealed that both groups exhibited the same profile for gender (p = 0.367) and spherical equivalence (p = 0.068). But the AOFVD group had greater age (p = 0.003).

**Fig. 3:**
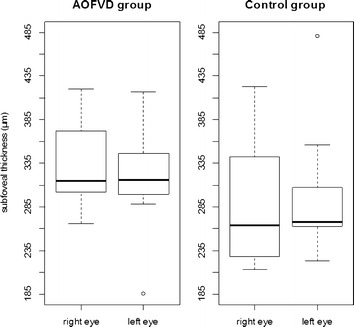
Boxplot of subfoveal thickness (µm) of both eyes from AOFVD group and control group

Comparison of subfoveal thickness and average of thickness between the groups were determined by analysis of variance (ANOVA) with repeated measures in that age, spherical equivalent and gender of the patients were considered as covariates (variables of control). The results revealed that the two groups (patients and controls) have similar measures of subfoveal thickness (p = 0.453) (Fig. 3) and average of thickness (p = 0.440).

In AOFVD group, men had a mean subfoveal thickness of 379.4 µm, ranging from 315 to 420 µm, with a standard deviation of 46.7 µm. The female patients had a mean subfoveal thickness of 305.0 µm, ranging from 186 to 420 µm, with a standard deviation of 58.4 µm (p = 0.023).

In control group, men had a mean subfoveal thickness of 320.0 µm, ranging from 226 to 481 µm, with a standard deviation of 74.1 µm. Women had a mean subfoveal thickness of 260.7 µm, ranging from 213 to 356 µm, with a standard deviation of 50.8 µm (p = 0.020).

**Fig. 4:**
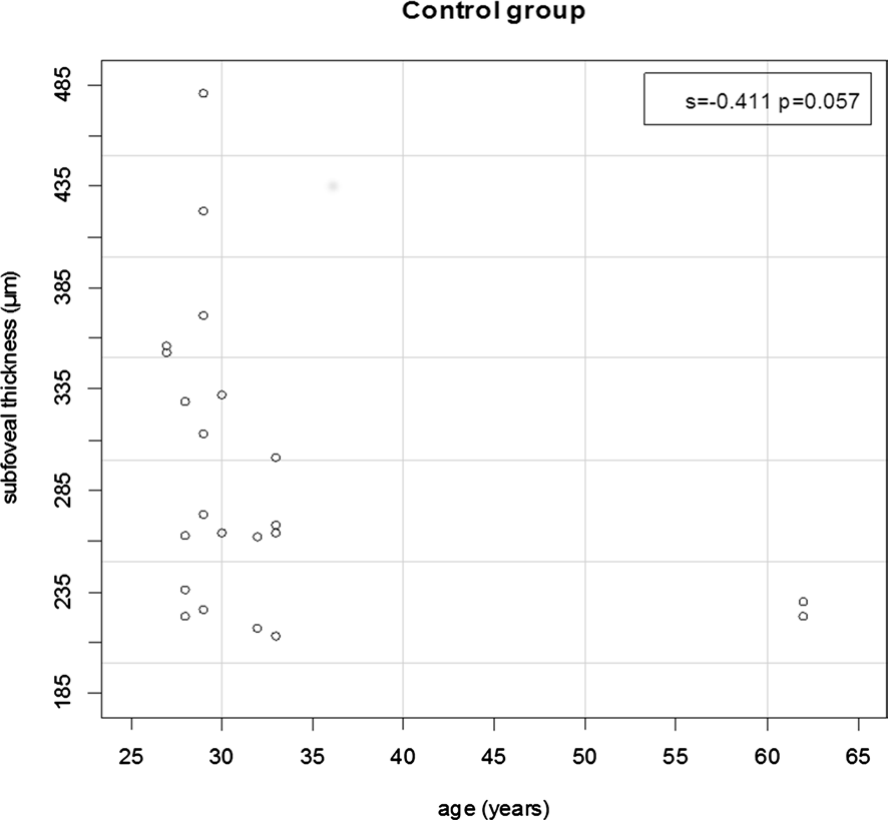
Two-dimensional scatter plot of subfoveal thickness (µm) and age (years) in control group

The correlation of subfoveal thickness with age was also investigated in this study. A trend (almost reached statistical significance) for a negative correlation between age and subfoveal thickness was observed only in control group (s = −0411; p = 0.057) (Fig. 4). This relation was confirmed by estimation of Spearman’s linear correlation coefficient (s). The coefficient lies in the −1 to +1 range, with values close to −1 or +1 indicating strong decreasing or increasing correlation between the pair of correlated numeric variables, respectively.

**Fig. 5:**
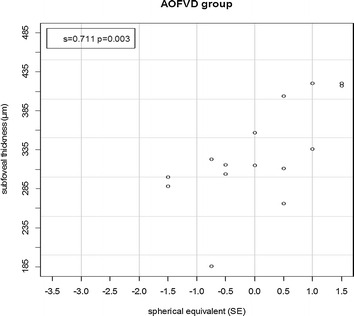
Two-dimensional scatter plot of subfoveal thickness (µm) and spherical equivalence (SD) in AOFVD group

Proportional correlation between spherical equivalent and subfoveal thickness was noted only in AOFVD group. The lower the spherical equivalent, the lower the subfoveal thickness (s = 0.711; p = 0.003) (Fig. 5). No significant correlation between subfoveal thickness and spherical equivalent was found in control group (s = −071; p = 0.8051).

## Discussion

AOFVD is a bilateral condition but may present with bilateral asymmetry. AOFVD is characterized by the accumulation, fragmentation and regression of yellowish subretinal material. The exact site of this vitelliform material has now been identified using high-definition OCT as situated between the interface of the internal and external segments of the photoreceptors and retinal pigment epithelium [2, 4]. Diagnosis is reached by complete ophthalmologic exam disclosing characteristic fundoscopic aspect, in addition to autofluorescence testing, fluorescein angiography, EOG and OCT [1].

**Fig. 6:**
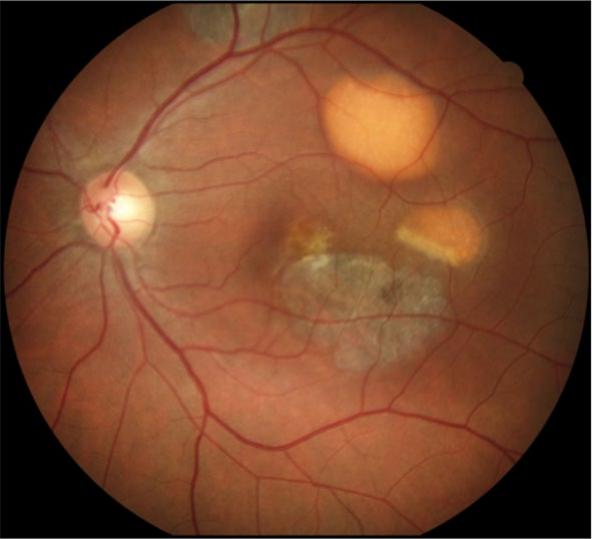
Retinography of a patient with multifocal vitelliform lesions from AOFVD group. Retinography of a patient from AOFVD group, showing multifocal vitelliform lesions not restricted to the macular region and different evolutionary stages, from classic vitelliform to atrophic

In distinguishing AOFVD from Best disease, despite the similar appearance of the vitelliform lesions, the EOG values is severely abnormal in Best disease, while in AOFVD it has been documented to range from normal to slightly subnormal. Best disease usually affects children or adolescents (3–15 years old) and the vitelliform lesion is placed in the macular region [1]. In this article, the age range of patients in AOFVD group was 33–62 years, some patients showed multifocal vitelliform lesions (Fig. 6) and also some of them have brought normal EOG exams.

Recent advances in SD-OCT allow highly detailed visualization of the choroid. Particularly through the use of EDI-OCT, the image quality of choroidal structures has improved substantially, enabling more accurate measurement of thickness [2, 7–9]. In the literature analyzed, mean choroidal thickness in the subfoveal region was 287.6 µm, with a standard deviation of 75.5 µm, showing high correlation in both eyes, decreasing in both sides of the fovea [8, 9].

Many authors have suggested that the choroid is involved in the pathogenesis of a variety of different ocular diseases. Evidence has shown significant increase in choroidal thickness in many diseases that involve the accumulation of fluid, associated with venous dilation and vascular hyperpermeability, such as CSC and PCV [2, 5, 6]. In AOFVD, the increased choroidal thickness most likely stems from an increase in circulation and vascular dilation in the choroid. This assumption is consistent with studies involving indocyanine green, which revealed diffuse intrachoroidal leakage in both eyes, even when only one eye had clinically demonstrable vitelliform deposits [2].

Both groups (AOFVD and controls) had similar measures of subfoveal thickness (p = 0.453) and average of thickness (p = 0.440). These data don’t corroborate the findings of other authors, that showed greater choroidal thickness in patients with AOFVD than healthy individuals [2, 3, 10] and also bigger than some diseases such as ARMD (reduced choroidal thickness) [2].

Men had statistically thicker subfoveal choroid than women in AOFVD group and also in control group. This information has been previously reported in some articles, which have also described, besides thicker choroid, greater retinal thickness in men [11], implying the existence of a hormonal influence. However, this finding contradicts the results of other studies [4, 9], which failed to find a correlation between choroidal thickness and sex.

In this article, a positive correlation was found between spherical equivalence and choroidal thickness only in AOFVD group. In the literature, choroidal thickness generally decreases with more negative spherical equivalent and increasing axial length [8, 11–14]. However, Wei et al. [2], found no statistical significance when refractive error exceeded −1.00 SD to positive diopters direction, whereas choroidal thickness reduced in cases below −1.00 SD with more negative SE. Similar findings were reported by Coscas et al. [15], who found no relation between emmetropic eyes and mildly myopic eyes but a statistically thicker choroid between these groups and highly myopic eyes. In the study by Kim et al. [9], no correlation between these two variables was evident.

No relation between subfoveal thickness and age was found in AOFVD group, as well as the study of Querques et al. [4]. However, a tendency for a negative correlation between these two variables was noted in the control group, in line with the majority of authors [7, 9, 11–13, 16, 17], showing that choroidal thickness reduces with advancing age.

## Conclusion

The AOFVD group and the control group showed similar choroidal thickness, not corroborating with few articles already published on this subject. Not yet known completely which biochemical and vascular flow alterations of the choroid, and which functional RPE changes may play a role in the pathogenesis of this disease. Therefore, studies that evaluate the choroid qualitatively and quantitatively, through non-invasive technological methods, are being increasingly published.

Our study has limitations. First, our sample is limited as the studied disease is relatively rare. Second, the EOG exams had been documented in another hospital prior to this study. Third, it is known that choroidal thickness changes with age. Despite the average age was higher in AOFVD group, the range of age between individuals of the two groups were similar and the comparison of subfoveal thickness between the groups were corrected for age (age as a covariate).

EDI-OCT, incorporated in some SD-OCT devices, allows higher quality assessment of the choroid. In this article, choroidal thickness of patients with AOFVD, a rare disease with a not fully understood pathogenesis, was assessed.

## Abbreviations

AOFVD: adult-onset foveomacular vitelliform dystrophy
ARMD: age-related macular degeneration
CSC: central serous chorioretinopathy
EDI-OCT: enhanced depth imaging optical coherence tomography
mm: millimeter
OCT: optical coherence tomography
PCV: polypoidal choroidal vasculopathy
RPE: retinal pigment epithelium
SD-OCT: spectral domain optical coherence tomography
µm: micrometer
SE: spherical equivalent
SD: spherical diopters
EOG: electrooculogram
